# Preparation of Antioxidant Peptides Derived From Quinoa Protein and Evaluation of Their Antioxidant Activity Through a D‐Galactose‐Induced Aging Mice Model

**DOI:** 10.1002/fsn3.71970

**Published:** 2026-05-31

**Authors:** Dandan Gao, Yuxuan Zhang, Hongxin Ma

**Affiliations:** ^1^ Key Laboratory of Biotechnology and Bioengineering of State Ethnic Affairs Commission Northwest Minzu University Lanzhou P. R. China; ^2^ China‐Malaysia National Joint Laboratory, Biomedical Research Center Northwest Minzu University Lanzhou P. R. China; ^3^ College of Life Science and Engineering Northwest Minzu University Lanzhou P. R. China

**Keywords:** anti‐aging effect, antioxidant activity, D‐galactose‐induced aging model, quinoa protein peptides

## Abstract

Quinoa is rich in essential nutrients and bioactive compounds. In this study, six proteases (Neutralase, Papain, Alcalase, Pepsin, Flavourzyme, Trypsin) were used to prepare quinoa protein peptides (QPP), and their antioxidant activity was assessed via in vitro chemical assays and a D‐galactose‐induced aging mouse model. Pepsin‐hydrolyzed QPP showed the highest in vitro antioxidant activity, in which essential amino acids accounted for 55.37% of the total amino acids. In vivo, QPP ameliorated organ index decline and pathological damage in the liver, kidney, and lung of aging mice, increased serum and liver SOD, CAT, GSH‐Px, and T‐AOC activities, reduced MDA levels, and upregulated the mRNA and protein expression of hepatic SOD1, SOD2, CAT, and GSH1 (*p* < 0.05). This study identifies pepsin as the optimal protease for preparing QPP and highlights QPP's potential as a natural anti‐aging agent, providing a theoretical basis for quinoa's high‐value application in functional foods.

## Graphical abstract

Quinoa protein peptides (QPP) were prepared via pepsin hydrolysis and showed strong in vitro antioxidant activity. In D‐galactose‐induced aging mice, QPP alleviated organ damage, enhanced antioxidant enzyme activities, and upregulated hepatic antioxidant gene and protein expression. QPP is a promising natural anti‐aging ingredient for functional foods.

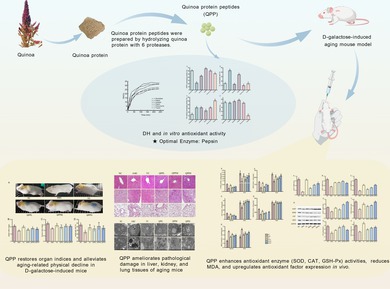

## 1 Introduction

*Chenopodium quinoa Willd*., commonly referred to as a pseudo‐cereal, has been extensively studied for its high‐quality protein content, which ranges from 12% to 23% (He et al. 2022). Research indicates that quinoa's protein content surpasses that of conventional cereal crops such as rice, barley, and corn (Pereira et al. 2019). Moreover, quinoa contains significantly higher levels of essential amino acids than rice (Shen et al. 2021). Due to its exceptional nutritional profile, quinoa has been recognized by the FAO (Van de Vondel et al. 2020) as a non‐GMO nutritional food and designated as “nutritional gold.” As a complete protein source, quinoa holds considerable potential for applications in both the food industry and nutraceutical development.

There is a growing body of research focused on the biological activities of protein hydrolysates and peptides derived from quinoa. Quinoa protein hydrolysates show significant potential as a source of multifunctional bioactive peptides, exhibiting antidiabetic (Mudgil et al. 2020), antihypertensive (Fan et al. 2023; Guo et al. 2020), hypolipidemic (Cao et al. 2020), antimicrobial, anticarcinogenic (Romero‐Benavides et al. 2023), anti‐inflammatory (Capraro et al. 2021), and antioxidant properties (Olivera‐Montenegro et al. 2020). However, most studies on quinoa‐derived antioxidant peptides have been limited to in vitro models, and further investigation is required to validate their in vivo antioxidant effects.

Aging is closely associated with increased endogenous production of free radicals. The persistent overproduction of free radicals in the body can lead to oxidative damage and progressive degeneration of biological systems (Halldorsdottir et al. 2014). Bioactive peptides exhibiting antioxidant properties can effectively neutralize excessive reactive oxygen species (ROS), thereby maintaining cellular redox homeostasis and protecting the structural and functional integrity of cells and mitochondria, which play a crucial role in delaying aging and preventing chronic diseases (Gawlik‐Dziki et al. 2013). In recent years, antioxidant peptides generated through enzymatic hydrolysis and microbial fermentation have emerged as a research focus due to their stable antioxidant activity, high safety profile, efficient digestibility and absorption, absence of toxic side effects, and wide availability (Rizzello et al. 2016). Numerous studies have successfully identified antioxidant peptides from food protein hydrolysates, including those derived from cottonseed (Gao et al. 2010), chicken serum (Gao et al. 2020), and mung bean (Xia et al. 2020).

To date, preclinical studies have demonstrated that antioxidant peptides confer protective effects against D‐galactose (D‐gal) induced aging in mice, effectively attenuating age‐related phenotypes. Among various sources, antioxidant peptides derived from quinoa protein have attracted increasing attention due to their natural origin and potential safety; however, research on these peptides remains limited. In contrast to the in vivo validation commonly performed for other antioxidant peptides, the antioxidant activity of quinoa‐derived peptides has primarily been assessed using in vitro chemical assays, which do not fully reflect their biological efficacy in vivo (Xie et al. 2008). Consistent with this, findings from previous studies confirm that quinoa protein‐derived antioxidant peptides exhibit strong in vitro antioxidant capacity, underscoring the necessity for further in vivo studies to validate their functional effectiveness (Guo et al. 2021). Accordingly, the in vivo anti‐aging effect and underlying molecular mechanism of quinoa protein peptides (QPP) still need to be systematically elucidated.

Therefore, this study used six proteases to prepare QPP and screened the optimal protease by in vitro antioxidant evaluation. The anti‐aging activity and mechanism of QPP were further investigated using a D‐gal‐induced aging mouse model. Results showed that pepsin‐hydrolyzed QPP possessed the highest in vitro antioxidant activity, and QPP significantly improved oxidative stress, organ damage, and immune organ atrophy in aging mice by upregulating hepatic antioxidant genes and proteins. This study confirms the promising anti‐aging potential of QPP and provides a theoretical basis for the high‐value application of quinoa in functional anti‐aging foods.

## 2 Materials and Methods

### 2.1 Materials and Reagents

Quinoa protein purchased from Xi'an Tech Biotechnology Co. Ltd. (Shanxi, China). Neutralase, Papain, Alcalase, Pepsin, Flavourzyme, Trypsin, o‐Phthalaldehyde (OPA), salicylic acid, potassium ferricyanide and vitamin C (VC) were obtained from Meilunbio Biological Reagent Co. Ltd. (Dalian, China). 1,1‐diphenyl‐2‐picrylhydrazyl (DPPH) and D‐gal were sourced from Sigma‐Aldrich Chemical Co. Ltd. (St. Louis, USA). All other reagents were of analytical grade and purchased from Sinopharm Chemical Reagent Co. Ltd. (Beijing, China). Enzyme‐linked immunosorbent assay (ELISA) kits for superoxide dismutase (SOD), catalase (CAT), malondialdehyde (MDA), glutathione peroxidase (GSH‐Px), and total antioxidant capacity (T‐AOC) were acquired from Solarbio Biological Reagent Co. Ltd. (Beijing, China).

### 2.2 Preparation of Antioxidant Peptides From Quinoa Protein

Five grams of quinoa protein was dissolved in 100 mL of distilled water and heated at 95°C for 10 min in a water bath. The solution was subsequently cooled to the optimal temperature for enzymatic digestion (as specified in Table 1), and adjusted to the optimal pH using either 1 mol/L NaOH or 1 mol/L HCl, supplemented with 5000 U/g of protease, and incubated for 4 h to facilitate complete enzymatic hydrolysis. The reaction was terminated by heating at 95°C for 10 min in a water bath, followed by centrifugation at 8000 r/min for 10 min using an X1R refrigerated centrifuge (Thermo Fisher Scientific, USA) (Xu et al. 2019). The supernatant was collected and freeze‐dried using an LGJ‐20F vacuum freeze‐dryer (Songyuan Huaxing Technology Development Co. Ltd., Beijing, China) and stored at −20°C until further analysis.

**TABLE 1:** Hydrolysis conditions of different proteases.

Protease	Substrate concentration (%)	pH	Temperature (°C)	Time (h)
Neutralase	5	7.0	45	4
Papain	5	7.5	55	4
Alcalase	5	8.5	60	4
Pepsin	5	2.0	40	4
Flavourzyme	5	7.0	50	4
Trypsin	5	8.5	50	4

Based on protease screening, pepsin was selected as the optimal enzyme for in vivo experiments. The specific hydrolysis conditions were: substrate concentration 5% (w/v), pH 2.0, temperature 40°C, hydrolysis time 4 h, enzyme dosage 5000 U/g.

### 2.3 Determination of the Degree of Hydrolysis (DH)

The degree of hydrolysis was determined using the OPA method (Intiquilla et al. 2018). A 1 μL aliquot of quinoa protein hydrolysate was diluted 1:1000 with deionized water. Then, 40 μL of this dilution was mixed with 300 μL of OPA reagent, vortexed thoroughly, and incubated for 2 min at room temperature. The absorbance was measured at 340 nm using a UV spectrophotometer (Unico Instrument Co. Ltd., Shanghai, China). A standard curve was constructed using serine as the reference standard. A stock solution of serine (0.8 mg/L) was serially diluted to concentrations of 0.40, 0.20, 0.10, 0.05, and 0.01 mg/mL, and the absorbance values were plotted against concentration to generate the standard curve. Based on the regression equation derived from the serine standard curve, the concentration of free amino groups in the hydrolysate was calculated. The DH was then determined according to the following equation:

(1) $$\mathrm{DH}=\frac{C}{N}\times 100$$

where DH is the degree of hydrolysis (%); C is the free amino group concentration in the hydrolysate (mg/mL); and N is the nitrogen content of the hydrolysate (mg/mL). The N was determined using the Kjeldahl method according to the standard procedure of AOAC 920.87.

### 2.4 In Vitro Antioxidant Activity Assay

#### 2.4.1 Determination of the DPPH Radical Scavenging Ability

Two milliliters of QPP samples at a concentration of 1.0 mg/mL were mixed with 2 mL of DPPH solution (0.2 mM) and reacted at room temperature for 30 min in the dark. The absorbance of the mixture was measured at 517 nm using a Multiskan GO multifunction microplate reader (Thermo Fisher Scientific, USA). Control 1 consisted of 2.0 mL of DPPH solution and 2.0 mL distilled water, Control 2 consisted of 2.0 mL of sample solution and 2.0 mL of distilled water, and the blank group was set as distilled water (Lu et al. 2019). The DPPH radical scavenging activity was calculated according to the following equation:

(2) $$E=1-\frac{A_{1}-A_{2}}{A_{0}}\times 100$$

where *E* is the DPPH radical scavenging rate (%); *A*_1_ is the absorbance of control 1; *A*_2_ is the absorbance of control 2; and *A*_0_ is the absorbance of the mixture of distilled water and DPPH.

#### 2.4.2 Determination of the O_2_ ^−^ Radical Scavenging Activity

All chemicals and samples were dissolved in Tris–HCl buffer (50 mmol/L, pH 8.0). Superoxide anion radicals were generated in a reaction mixture containing nitrotetrazolium blue chloride (NBT) (0.5 mL, 300 μmol/L), nicotinamide adenine dinucleotide hydrogen (NADH) (0.3 mL, 468 μmol/L), and phenazinemethosulfate (PMS) (0.5 mL 60 μmol/L). After vigorous agitation, 1.5 mL of quinoa protein hydrolysate was added to the mixture and incubated at 25°C for 5 min. For the control group, Tris–HCl buffer (50 mmol/L, pH 8.0) was used in place of quinoa protein hydrolysate, with all other conditions kept identical. The absorbance of the reaction mixture was measured at 560 nm (Xing et al. 2016). The O_2_^−^ radical scavenging activity was calculated using the following equation:

(3) $$E=\left(1-\frac{A_{0}}{A}\right)\times 100$$

where *E* is the O_2_^−^ radical scavenging rate (%); *A*_0_ is the absorbance of the blank; and *A* is the absorbance of the sample.

#### 2.4.3 Determination of the Hydroxyl Radical (•OH) Scavenging Activity

One milliliters of a 1.0 mg/mL QPP solution was mixed with 1.0 mL of o‐phenanthroline ethanol solution (0.75 mM) and 1.0 mL of FeSO_4_ solution (0.75 mM), and the mixture was incubated in a water bath at 37°C for 30 min. Subsequently, 1.0 mL of H_2_O_2_ (0.01%) and 2.0 mL of PBS (pH 7.4) were added. After thorough mixing, the reaction mixture was incubated at 37°C for an additional 15 min. Following cooling to room temperature, the absorbance of the sample solution was measured at 510 nm (Xia et al. 2020). The •OH scavenging activity was calculated using the following equation:

(4) $$E=\frac{A_{0}-A}{A_{0}}\times 100$$

where *E* is the •OH radical scavenging activity (%); *A* denotes the absorbance of the sample solution; and *A*_0_ denotes the absorbance of the blank solution.

#### 2.4.4 Determination of Potassium Ferricyanide Reducing Antioxidant Power (PFRAP)

One milliliters of a 1.0 mg/mL QPP solution was added to 2.5 mL of phosphate buffer solution (PBS, pH 6.6) and 2.5 mL of K_3_Fe(CN)_6_ solution. The volume was adjusted to 10 mL with distilled water, and the mixture was incubated at 50°C for 30 min. After cooling to room temperature (25°C), the reaction was terminated by adding 2.5 mL of 10% trichloroacetic acid. The dispersion was centrifuged at 5000 r/min, and 2 mL of the supernatant was transferred to a new tube. To this, 2 mL of deionized water and 0.4 mL of 1% FeCl_3_ solution were added, and the mixture was allowed to react for 15 min at room temperature. The absorbance was measured at 700 nm immediately after the reaction (Phinyo et al. 2022). The PFRAP was calculated using the following equation:

(5) $$E=\frac{A_{1}-A_{0}}{A_{2}-A_{0}}\times 100$$

where *E* is the PFRAP (%); *A*_0_ is the absorbance of the blank group; *A*_1_ is the absorbance of the sample group; *A*_2_ is the absorbance of the reference group (vitamin C).

### 2.5 Analysis of Amino Acid Composition of Quinoa Protein Hydrolysate

Twenty milligrams of QPP were accurately weighed and transferred into a 20 mL hydrolysis tube. Ten milliliters of 6 mol/L HCl 10 mL were added, followed by nitrogen purging with high‐purity nitrogen for 5 min. The hydrolysis tube was sealed and incubated at 110°C for 22 h in a thermostatic oven. After cooling to room temperature, 1 mL of the filtered hydrolysate was evaporated to dryness using a tube concentrator under reduced pressure. The residue was reconstituted in 2–5 mL of sample diluent, vortexed thoroughly, and sonicated in an ultrasonic cleaner to ensure complete dissolution. The solution was then filtered through a 0.22 μm membrane filter prior to analysis. Amino acid composition was determined using a L‐8900 automatic amino acid analyzer (Hitachi High‐Technologies Corporation, Tokyo, Japan) (Bai et al. 2022).

### 2.6 Animal Experimental Design

A total of 60 female Balb/c mice (specific‐pathogen free, 18–22 g, 5 weeks old) were obtained from the Laboratory Animal Center of Lanzhou University and housed at a controlled temperature of 24°C ± 2°C and relative humidity of 60% ± 5%, under a 12 h light/dark cycle. All animal procedures were conducted in accordance with the National Guidelines for the Care and Use of Laboratory Animals and were approved by the Animal Ethics Committee of Northwest Minzu University (approval number: xbmu‐sm‐2025101).

After a 7‐day acclimatization period, all mice were randomly divided into six groups (*n* = 10): a normal control group (NC), a D‐galactose‐induced aging model group (AMC), a low‐dose quinoa protein peptide group (QPPL, 200 mg/kg), a medium‐dose quinoa protein peptide group (QPPM, 400 mg/kg), a high‐dose quinoa protein peptide group (QPPH, 800 mg/kg), and a positive control group (VC, 200 mg/kg). The dose levels (200, 400, and 800 mg/kg) were selected based on preliminary experiments, previous literature, and commonly used doses in D‐galactose‐induced aging mouse models. In the preliminary experiment, six dose gradients (100, 200, 400, 800, 1200, and 1600 mg/kg) were tested for 4 weeks, with body weight, liver SOD activity, and MDA content as the main evaluation indicators. The 100 mg/kg group showed weak antioxidant effects, while the 1200 and 1600 mg/kg groups showed no further improvement in efficacy but mild reduction in food intake. The dose range of 200–800 mg/kg exhibited significant dose‐dependent antioxidant activity and favorable safety. Therefore, these three doses were selected for the formal study.

Except for the NC group, which received an equivalent volume of normal saline, all other groups were subcutaneously injected with D‐gal (1000 mg/kg·day) at the nape of the neck for 10 consecutive weeks to establish the aging model. Starting from the fourth week of modeling, mice in the QPPL, QPPM, QPPH, and VC groups were administered the corresponding treatments by daily gavage, while the NC and AMC groups received an equal volume of distilled water. During the entire experiment, body weights were recorded every 3 days, and food and water were available ad libitum. At 24 h after the last administration, all mice were anesthetized and humanely euthanized according to ethical protocols. The liver, lung, and kidney tissues were quickly collected and rinsed with ice‐cold 0.9% normal saline. Half of each tissue was fixed for histological examination, and the other half was stored for biochemical detection and Western blot analysis. Blood samples and the remaining half of the liver tissue were collected for the determination of cytokine levels by ELISA and the evaluation of serum enzyme activities. The spleen and thymus were isolated and weighed to calculate the organ indices.

### 2.7 Observation of the General Condition of the Mice

Throughout the experiment, the general appearance and behavioral condition of the mice and behavioral condition were recorded weekly, including food and water intake, coat color and thickness, and characteristics of urinary and fecal output. Body weight was recorded weekly as an indicator of overall health, and the weight gain rate was calculated using the following formula:

(6) $$E=\frac{M-m}{m}\times 100$$

where *E* is the weight gain rate (%); *M* is the final body weight (g); and *m* is the initial body weight (g).

### 2.8 Measurement of Organ Weight Indices

At the end of the study, the thymus and spleen were carefully dissected, rinsed with ice‐cold physiological saline, blotted dry, and immediately weighed. The thymus and spleen indices were calculated using the following formula:

(7) $$E=\frac{m}{M}$$

where *E* is the organ index (mg/g); *m* is the organ weight (mg); and *M* is the body weight of mice (g).

### 2.9 Determination of SOD, CAT, MDA, GSH‐Px, and T‐AOC in Serum and Liver

The liver was rapidly excised, weighed, and immediately homogenized in nine volumes of cold 0.9% normal saline (4°C) using an IKA Ultra Turrax T18 Basic homogenizer (IKA‐Werke GmbH & Co. KG, Staufen, Germany) to prepare a 10% (w/v) tissue homogenate. The homogenate was subsequently centrifuged at 4000 rpm for 10 min at 4°C, and the supernatant was collected and stored at −80°C until analysis. Blood samples were collected from the orbital sinus of the mice, and serum was separated by centrifugation at 3000 rpm for 10 min at 4°C. Both the supernatant and serum were used to measure the levels of SOD, CAT, MDA, GSH‐Px, and T‐AOC (BC5156, BC0205, BC0025, BC1195, BC1315, Beijing Solarbio, Beijing, China) in liver tissue and serum, respectively, according to the manufacturer's instructions, using commercially available assay kits.

### 2.10 Hematoxylin and Eosin (H&E) Staining

The liver, kidney, and lung specimens were fixed in 4% formalin and subsequently embedded in paraffin. Paraffin‐embedded sections (3.5 μm thick) were subjected to H&E staining. Histological images were then captured using a light microscope (Olympus Corporation, Tokyo, Japan).

### 2.11 Transmission Electron Microscopy (TEM) Analysis

The liver specimens were rinsed with saline, cut into small pieces (1 mm^2^), immediately fixed in 2.5% glutaraldehyde fixative, post‐fixed in 1% osmium tetroxide solution, dehydrated through a graded ethanol series, embedded in epoxy resin, sectioned, and double stained with uranyl acetate and lead citrate.

### 2.12 RNA Extraction and Quantitative by Quantitative Real‐Time PCR (qRT‐PCR)

Total RNA was extracted from liver tissue using a total RNA extracted kit (G3074‐200T, Servicebio, Wuhan, China). The concentration and purity of the RNA were determined using a Varioskan LUX multimode microplate reader (Thermo Fisher, Massachusetts, USA). One microgram of total RNA was reverse transcribed into complementary DNA using a RT First Strand cDNA Synthesis Kit (G3331, Servicebio, Wuhan, China). qRT‐PCR was performed in a 15 μL reaction mixture containing 7.5 μL 2×SYBR Green qPCR Master Mix (Low ROX) (Servicebio, Wuhan, China), 1.5 μL 2.5 μM gene‐specific primers, 2 μL of reverse transcription product, and 4 μL RNase‐free dH_2_O. Amplification was carried out on a CFX Real‐Time PCR Detection System (Bio‐Rad, Hercules, USA). Fluorescence signals were monitored at each cycle to track amplification in real time. The comparative cycle threshold (Ct) method was used to calculate relative gene expression levels. The housekeeping gene GAPDH was used as an internal control to normalize variations in RNA quantity and quality across samples. Fold changes in gene expression were calculated using the ∆∆CT method according to the formula 2^−∆∆CT^ and presented relative to the control group. Gene‐specific primers for SOD1, GADPH, Catalase, and GSH1 were synthesized by Servicebio (Wuhan, China). Primer sequences are listed in Table 2 (Yang et al. 2022).

**TABLE 2:** Primer sequences.

Gene name	Forward primer (5′ to 3′)	Reverse primer (3′ to 5′)
SOD1	ATGTGACTGCTGGAAAGGACG	CGCAATCCCAATCACTCCAC
CAT	CCAGCGACCAGATGAAGCAGT	CAGGAATCCGCTCTCTGTCAA
GSH1	TATCTGAACCTGTCCGAGAAGCA	TCGTCGTCCTCTGAGCATTTG
GAPDH	CCTCGTCCCGTAGACAAAATG	TGAGGTCAATGAAGGGGTCGT

### 2.13 Western Blot Analysis

Total protein was extracted from spleen tissue of mice in each group using a total protein extraction maxi kit (BC3710, Beijing Solarbio, Beijing, China), and protein concentration was determined using a BCA Protein Assay Kit (PC0020, Beijing Solarbio, Beijing, China). Proteins were separated by sodium dodecyl sulfate‐polyacrylamide gel electrophoresis (SDS‐PAGE) and subsequently transferred onto nitrocellulose membranes. The membranes were blocked with 5% skim milk and incubated with primary antibodies against SOD1, SOD2, catalase, GSH1 (Servicebio, Wuhan, China; 1:1000), and GAPDH (Servicebio, Wuhan, China; 1:5000), followed by incubation with corresponding secondary antibodies. Protein bands were visualized using the ECL Western Blotting Substrate (Bio‐Rad, Hercules, USA) on an Image Quant LAS 4000 mini imager (GE Healthcare, Pittsburgh, USA) (Sun et al. 2022).

### 2.14 Statistical Analysis

In the present study, all results were derived from three or more independent replicates and are expressed as mean ± standard deviation (SD). Statistical significance was assessed using one‐way analysis of variance (ANOVA) followed by Tukey's multiple comparisons tests. Data were analyzed using SPSS software (version 25.0; IBM, Chicago, USA), and a *p* value of less than 0.05 was considered statistically significant.

## 3 Results

### 3.1 Evaluation of DH and Antioxidant Capacity of Quinoa Hydrolysates From Six Proteases

**FIGURE 1:**
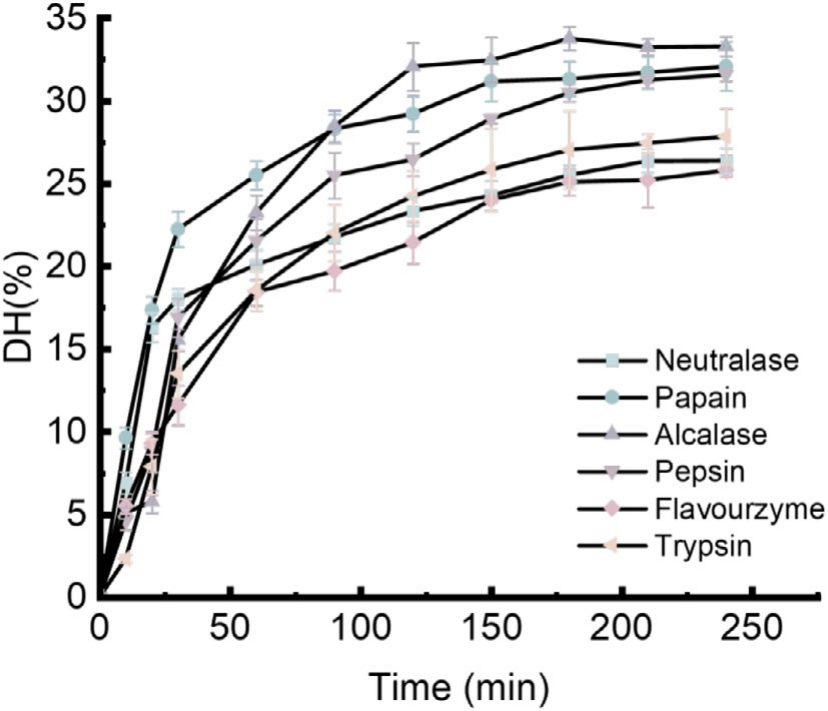
DH of hydrolysates from six proteases. Data shown were the mean ± SD (*n* = 5).

The antioxidant peptides from quinoa were prepared by hydrolyzing quinoa protein using six proteolytic enzymes: Neutralase, Papain, Alcalase, Pepsin, Flavourzyme, and Trypsin. As shown in Figure 1, during the initial 0–90 min of hydrolysis, the DH increased rapidly for all enzymes. Notably, Alcalase and Papain exhibited a more pronounced increase in the early phase, with Alcalase reaching a relatively high DH level by approximately 90 min. From 90 to 210 min, the DH continued to rise, although the rate of increase gradually slowed. Alcalase maintained the highest DH value throughout this period, while Papain and Neutralase also showed sustained increases. Beyond 210 min, the DH values plateaued for all protease, indicating that the hydrolysis reaction had entered a stable phase. The stabilization of DH suggests that the enzymatic reactions approached completion or reached equilibrium, with Alcalase consistently achieving the highest hydrolysis efficiency.

**FIGURE 2:**
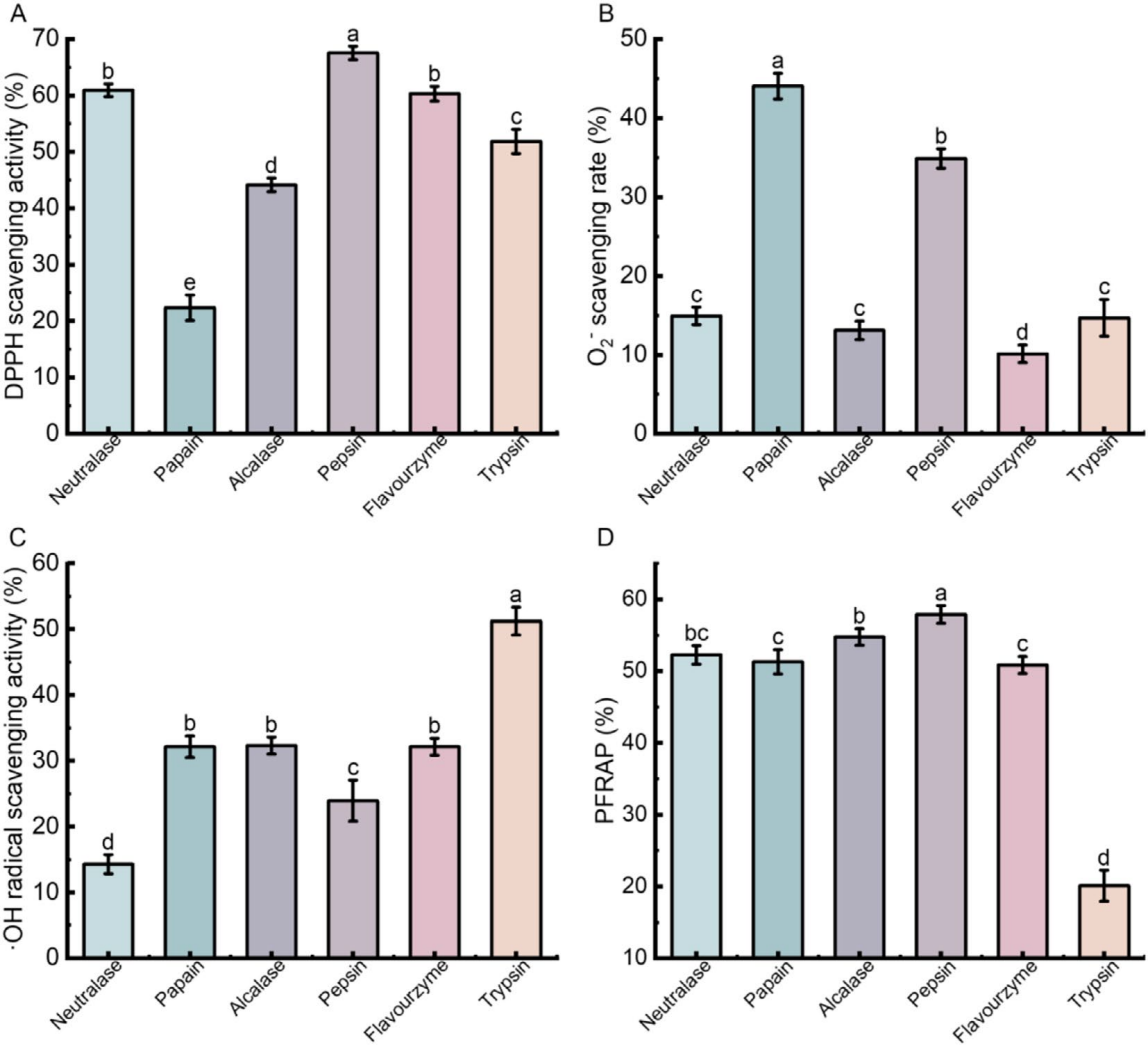
In vitro antioxidant activity of hydrolysates from six proteases. (A) DPPH radical scavenging activity; (B) O_2_^−^ scavenging rate; (C) ·OH radical scavenging activity; (D) PFRAP. Data shown were the mean ± SD (*n* = 5). Mean values with different lowercase letters in the same bar graph are significantly different (*P* < 0.05) according to Duncan's multiple range test; the same as in other figures.

The in vitro antioxidant activity of six hydrolysates was evaluated, and the results are presented in Figure 2. Figure 2A shows the DPPH radical scavenging activity of the six protease‐derived hydrolysates. DPPH radical is a stable free radical commonly used in antioxidant assays due to its stability in organic solvents; upon interaction with antioxidants, the unpaired electrons of DPPH are reduced, leading to a measurable decrease in absorbance at 517 nm (Shafiq et al. 2022). Among the tested hydrolysates, pepsin‐derived hydrolysate exhibited the highest scavenging activity (67.54% ± 1.19%), likely attributable to pepsin's substrate specificity during protein hydrolysis, which generates small peptides with enhanced antioxidant potential. Neutralase and Flavourzyme hydrolysates demonstrated moderate activities with scavenging rates of 60.94% ± 1.13% and 60.31% ± 1.31%, respectively. In contrast, Papain hydrolysate showed the lowest DPPH radical clearance rate (22.35% ± 2.25%).

The O_2_^−^ scavenging activities of the six protease‐derived hydrolysates are shown in Figure 2B. O_2_^−^ can react with hydroxyl groups to form harmful substances that induce lipid peroxidation, leading to mitochondrial dysfunction and cellular damage (Raghavan and Kristinsson 2009). Among the tested hydrolysates, papain exhibited the highest scavenging activity at 44.04% ± 1.62%, followed by Pepsin at 34.88% ± 1.24%, while Flavourzyme showed the lowest activity at 10.13% ± 1.11%.

The ·OH radical scavenging activities of the six protease‐derived hydrolysates are shown in Figure 2C. Hydroxyl radicals are highly reactive and oxidizing species that can damage erythrocytes and degrade biological macromolecules such as DNA, cell membranes, and polysaccharides (Chen et al. 2011). Among the tested hydrolysates, Trypsin exhibited the highest scavenging activity against ·OH radicals, reaching 51.25% ± 2.12%. The activities of papain, Flavourzyme, and Alcalase were comparable, ranging from 32.14% ± 1.59% to 32.32% ± 1.28%. Pepsin showed a moderate scavenging activity of 23.93% ± 3.13%, while neutralase displayed the lowest activity at 14.29% ± 1.81%.

The PFRAP values of the six protease‐derived hydrolysates are shown in Figure 2D. Reducing capacity reflects the ability of antioxidants to donate electrons, serving as an indicator of their electron‐donating potential. Among the tested hydrolysates, pepsin hydrolysate exhibited the highest reducing power at 57.9% ± 1.23%, followed by Alcalase hydrolysate at 54.75% ± 1.15%, while Trypsin hydrolysate showed the lowest activity at 20.1% ± 2.16%.

In summary, after a comprehensive evaluation of the antioxidant effects of hydrolysates generated by six proteases, Pepsin was selected as the optimal enzyme for the production of antioxidant peptides from quinoa protein, as its hydrolysate demonstrated strong in vitro antioxidant activity.

### 3.2 Amino Acid Composition of Pepsin Hydrolysate of Quinoa Protein

As shown in Table 3, the essential amino acid content of quinoa protein and pepsin hydrolysate accounted for 37.95% and 55.37% of the total amino acids, respectively. Certain amino acids such as Tyr, His, Phe, and Lys are commonly recognized for their antioxidant potential. Notably, His contains an imidazole ring capable of chelating metal ions, thereby inhibiting metal ion‐catalyzed or ‐promoted oxidation reactions, which enhances the antioxidant capacity of the resulting peptides (Liu et al. 2015). Gly is an amino acid with both acidic and basic functional groups in its molecule and serves as a key constituent of GSH, a potent antioxidant (Lu 2020). After pepsin hydrolysis of quinoa protein, hydroxylysine content decreased, while the levels of all other amino acids increased. Leu, Phe, and Arg were present at relatively high concentrations in the pepsin hydrolysate and are considered the primary amino acids contributing to the antioxidant peptides generated by this hydrolysis process. The enhanced antioxidant activity of these peptides is closely associated with their favorable amino acid composition.

**TABLE 3:** Analysis of amino acid composition of quinoa protein and pepsin hydrolysate.

Amino acids (mg/g)	Quinoa protein	Pepsin hydrolysate
Thr	49.32	630.42
Val	28.17	500.54
Met	34.51	188.48
Ile	33.85	832.39
Leu	39.99	1907.93
Phe	29.85	1315.24
Lys	29.99	827.46
Trp	10.25	260.12
Asp	56.52	487.52
Ser	34.85	833.55
Glu	85.99	501.36
Sar	19.67	91.89
Ala	22.73	308.94
Cit	17.52	189.14
Cys	10.16	69.43
Tyr	30.63	865.46
Hylys	29.92	1.49
Orn	35.36	114.81
His	15.86	416.17
Arg	37.8	1199.36
Gly	21.32	130.86
Total	674.26	11,672.56

### 3.3 Effects of QPP on the General Condition and Body Weight of D‐Gal‐Induced Aging Mice

**FIGURE 3:**
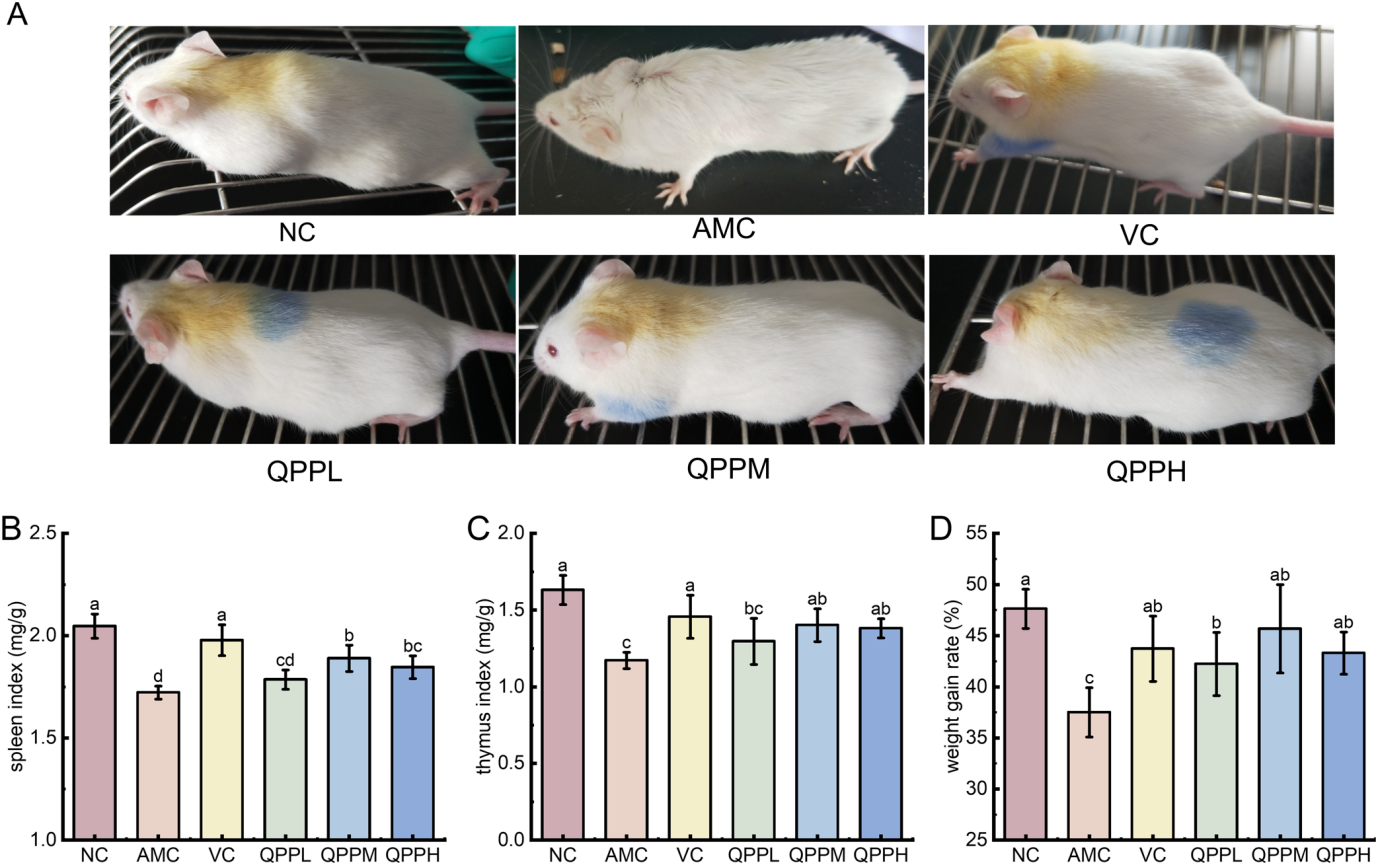
Effect of QPP on the general condition, weight gain rate, and organ coefficients of senescent mice induced by D‐gal. (A) The general condition of mice (B) Body weight gain; (C) Spleen index; (D) Thymus index. Data shown were the mean ± SD (*n* = 8).

During the experimental period, mice in the NC group appeared healthy and alert with smooth fur, alertness, and dense, smooth fur. In contrast, mice in the AMC displayed dull, yellowish, loose, and lusterless fur, hunched posture, loose skin, reduced appetite, body weight loss, slow movement, and signs of depression indicative of aging. Compared with the AMC group, mice treated with VC and quinoa protein‐derived antioxidant peptides showed gradual improvements in fur color, body shape, appetite, mental status, spontaneous activity, and response sensitivity. Their overall condition approached that of the NC group, as shown in Figure 3A.

Weight gain can serve as an indicator of overall health. As shown in Figure 3B, compared with the normal control group, the weight gain of the aging group injected with D‐gal was significantly lower (*p* < 0.05). In contrast, mice treated with QPP and VC exhibited significantly greater weight gain compared to the AMC group (*p* < 0.05). These results suggest that QPP may have the potential to improve the overall health status of D‐gal‐induced aging mice.

### 3.4 Effect of QPP on Organ Index in D‐Gal‐Induced Aging Mice

The thymus and spleen are critical immune organs involved in regulating immune responses. Age‐related decline in immune function is one of the primary hallmarks of aging (Uddin et al. 2010). Consequently, the spleen index is widely regarded as a biomarker of immunological status. As shown in Figure 3C,D, D‐gal administration led to a significant reduction in thymus and spleen indices, indicating that the modeling process induced marked atrophy of immune organs in mice. In contrast, treatment with QPP—particularly QPPM and QPPH—significantly reversed these reductions compared to the model group (*p* < 0.05), while QPPL exhibited only a modest improvement in organ indices. Overall, these findings demonstrate that administration of QPP and VC effectively mitigates age‐associated atrophy of the thymus and spleen, enhances immune function, and thereby contributes to delaying the aging to a certain extent.

### 3.5 Effects of QPP on D‐Gal‐Induced Oxidative Stress Injury in Serum and Liver

**FIGURE 4:**
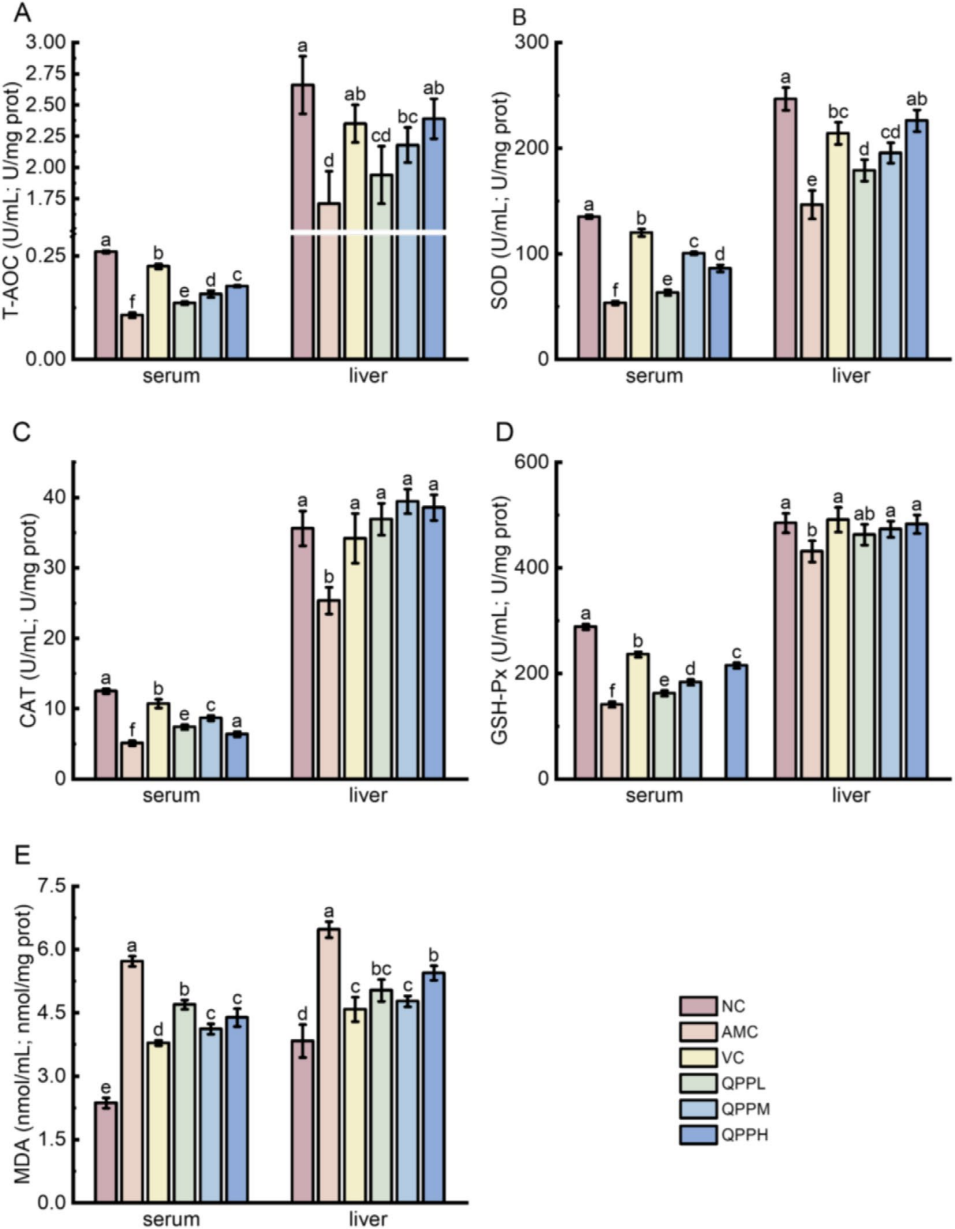
Effect of QPP on the activities of antioxidant indicators and the levels of MDA in the serum and liver of D‐gal‐treated mice. (A) The activities of T‐AOC; (B) SOD; (C) CAT; (D) GSH‐Px; (E) MDA. Data shown were the mean ± SD (*n* = 6).

To investigate whether QPP can alleviate oxidative stress in D‐gal‐induced mice, we evaluated the activities of key antioxidant enzymes (including SOD, CAT, and GSH‐Px) in both serum and liver tissues, along with T‐AOC and MDA levels. As shown in Figure 4, the activities of SOD, CAT, GSH‐Px, and T‐AOC in the serum and liver of the AMC group were significantly lower than those in the NC group (*p* < 0.05), while MDA levels in both serum and liver were markedly elevated compared to the NC group (*p* < 0.05). Administration of QPPL significantly reduced MDA concentration (*p* < 0.05), and led to significant improvements in serum SOD, GSH‐Px, and T‐AOC levels (*p* < 0.05). Moreover, treatment with medium‐ and high‐dose QPP (QPPM and QPPH) as well as VC resulted in significantly enhanced antioxidant enzyme activities (SOD, CAT, GSH‐Px, and T‐AOC) and reduced MDA levels in both serum and liver. Collectively, these results indicate that QPP supplementation effectively attenuates D‐gal‐induced oxidative stress and exerts substantial protective effects.

### 3.6 Effect of QPP on the Histomorphology of Mice

**FIGURE 5:**
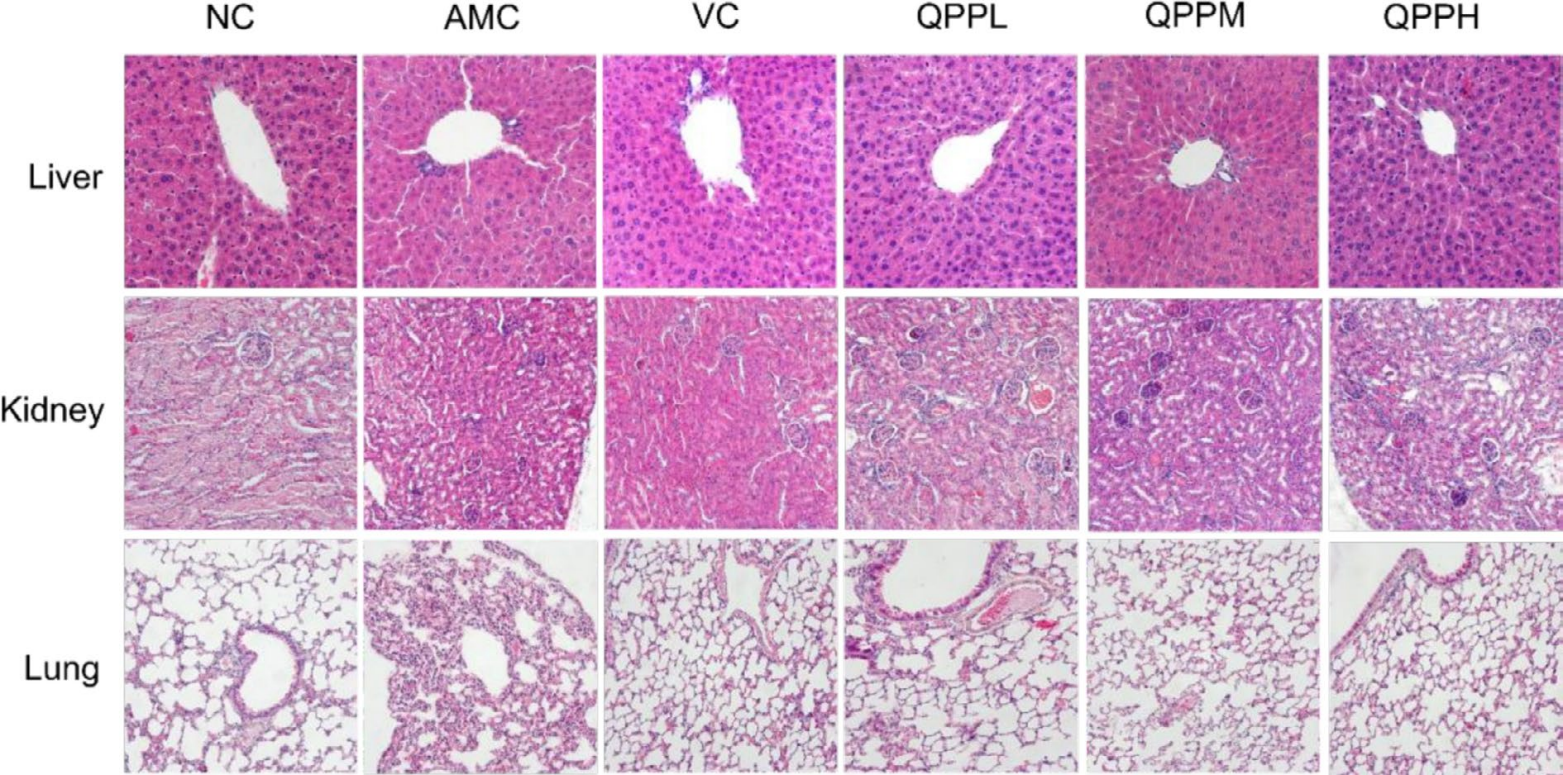
Pathological sections of mouse organs stained with HE (HE 400×).

As shown in Figure 5, hepatocytes in the NC group were predominantly binucleated and arranged in a plate‐like pattern radiating from the central vein. The hepatic sinusoidal spaces were moderate, and the hepatocytes exhibited well‐defined boundaries without signs of swelling or atrophy, with no obvious pathological lesions observed. Mild suspected amyloidosis was noted. In contrast to the AMC group, mice treated with VC, QPPL, QPPM, and QPPH showed substantially preserved hepatic architecture: cell arrangement was largely orderly, hepatic cords were relatively neat, hepatocytes exhibited only mild swelling, and nuclear structures were comparatively clear, indicating a trend toward normalization of liver histology. These findings suggest that QPP can alleviate hepatic histopathological changes in D‐gal‐induced aging model mice.

The glomerular endothelial cells in kidney tissue of mice in the NC group were uniform, with a moderate glomerular space, clearly defined structure of surrounding proximal tubules, normal and patent lumina, and tightly organized, well‐aligned tubular epithelial cells. In contrast, in the AMC group, some glomeruli exhibited swelling with loss of glomerular space, while others showed atrophy; the architecture of the adjacent proximal tubules was disorganized, tubular epithelial cells were swollen, the lumina were narrowed, nuclear density was increased, and cellular structures were indistinct. Compared with the AMC group, mice in the VC, QPPL, QPPM, and QPPH groups displayed a significantly greater number of glomeruli with preserved normal morphology and structural integrity, reduced renal capsule cavities, and no obvious basement membrane thickening. These findings indicate that quinoa protein antioxidant peptides can ameliorate renal injury in aging model mice induced by D‐gal.

The NC group exhibited uniform alveolar size, moderate septal thickness, well‐defined alveolar capsule structure, abundant capillaries in the alveolar septa, and no evident pathological alterations. In contrast, the AMC group displayed disorganized lung tissue architecture, including fused alveolar capsules, collapsed or absent alveoli, thickened and fragmented alveolar septa, interstitial and parenchymal fusion, loss of visible respiratory bronchiole structure, alveolar cell necrosis, and inflammatory cell infiltration. Compared with the AMC group, the VC, QPPL, QPPM, and QPPH groups showed marked histological improvement, characterized by more uniform alveolar size, clearly discernible alveolar sac structure, and only mild alveolar septal thickening and interstitial edema. These observations suggest that quinoa protein antioxidant peptides can mitigate pulmonary structural damage in D‐gal‐induced aging model mice.

### 3.7 Effects of QPP on Mouse Liver and Mitochondria

**FIGURE 6:**
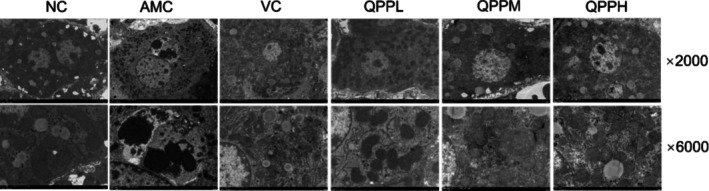
Ultrastructure of hepatocytes and mitochondria in different groups.

As shown in Figure 6, binucleated hepatocytes were observed in the NC group, with clearly defined nuclear structure, intact and distinct nuclear membrane, and uniformly distributed chromatin. The cytoplasmic architecture was well preserved, with mitochondria and ribosomes densely arranged around the nucleus, and numerous Golgi apparatuses exhibiting intact structures and smooth surfaces were visible in the lower portion of the field. Under high‐magnification imaging, mitochondrial morphology appeared normal, with clearly visible and structurally intact outer and inner membranes as well as cristae showing no signs of swelling or shrinkage.

In the AMC group, nuclei appeared rounded with clearly visible nuclear membranes and uniformly distributed chromatin; however, localized membrane ruptures were observed. The cytoplasmic structure was blurred and disintegrated, with increased mitochondrial electron density, disappearance of the endoplasmic reticulum, numerous lipid droplets of varying sizes, and an increased number of lysosomes. Under high‐magnification imaging, mitochondrial cristae were no longer discernible, and signs of solid contraction were evident. Moreover, mitochondria surrounded by lysosomes were present in the perinuclear region.

In the VC group, nuclei were rounded with clear nuclear membranes and uniformly distributed chromatin. The cytoplasmic structure was intact, with mitochondria and endoplasmic reticulum interspersed and clearly organized. Additionally, lipid droplets of varying sizes and lysosomes were scattered throughout the field. Under high‐magnification imaging, mitochondrial morphology was preserved, with moderate electron density; however, some mitochondria exhibited cristae lysis or discontinuities. The rough endoplasmic reticulum maintained structural integrity, and the ribosomes on its surface were clearly visible.

In the QPPL group, nuclei were structurally intact with clearly defined nuclear membranes and uniformly distributed chromatin. The cytoplasm contained numerous mitochondria with slightly increased electron density, and the endoplasmic reticulum was evenly distributed and morphologically well preserved. Under high‐magnification imaging, mitochondria appeared structurally intact, and the endoplasmic reticulum was largely intact, with only minor swelling and occasional lysis or fragmentation.

In the QPPM group, nuclei exhibited intact structure with clearly defined nuclear membranes and uniformly distributed chromatin. The cytoplasmic architecture was largely clear and well preserved, although mild scattering and focal blurring were occasionally observed in localized regions. Mitochondria and endoplasmic reticulum appeared structurally distinct and readily discernible. Additionally, scattered lysosomes and lipid droplets were present throughout the cytoplasm. Under high‐magnification imaging, mitochondria maintained structural integrity, with uniform membrane thickness and well‐preserved cristae. The endoplasmic reticulum remained intact and continuous, displaying consistent and moderate width.

In the QPPH group, nuclei within the field of view exhibited intact morphology, with clearly delineated nuclear membranes and uniformly distributed chromatin. The cytoplasmic structure appeared clear and homogeneously textured. Numerous mitochondria and endoplasmic reticulum were present, displaying preserved ultrastructural integrity and moderate electron density; scattered lipid droplets were observed in localized regions. Under high‐magnification imaging, the endoplasmic reticulum maintained structural continuity with consistent membrane thickness, while mitochondrial membranes and cristae remained largely intact. A small number of mitochondria showed evidence of complete fusion.

Electron microscopy revealed that hepatocytes in the NC group were abundant in organelles, with intact mitochondrial outer and inner membranes and clearly visible cristae. In contrast, hepatocytes and mitochondria in the AMC group exhibited marked ultrastructural damage. Compared to the AMC group, the VC, QPPL, QPPM, and QPPH treatment groups displayed varying degrees of structural improvement, indicating partial restoration of mitochondrial and cellular integrity.

### 3.8 Effect of QQP on SOD1, GSH1, and CAT mRNA Levels in Mouse Liver

**FIGURE 7:**
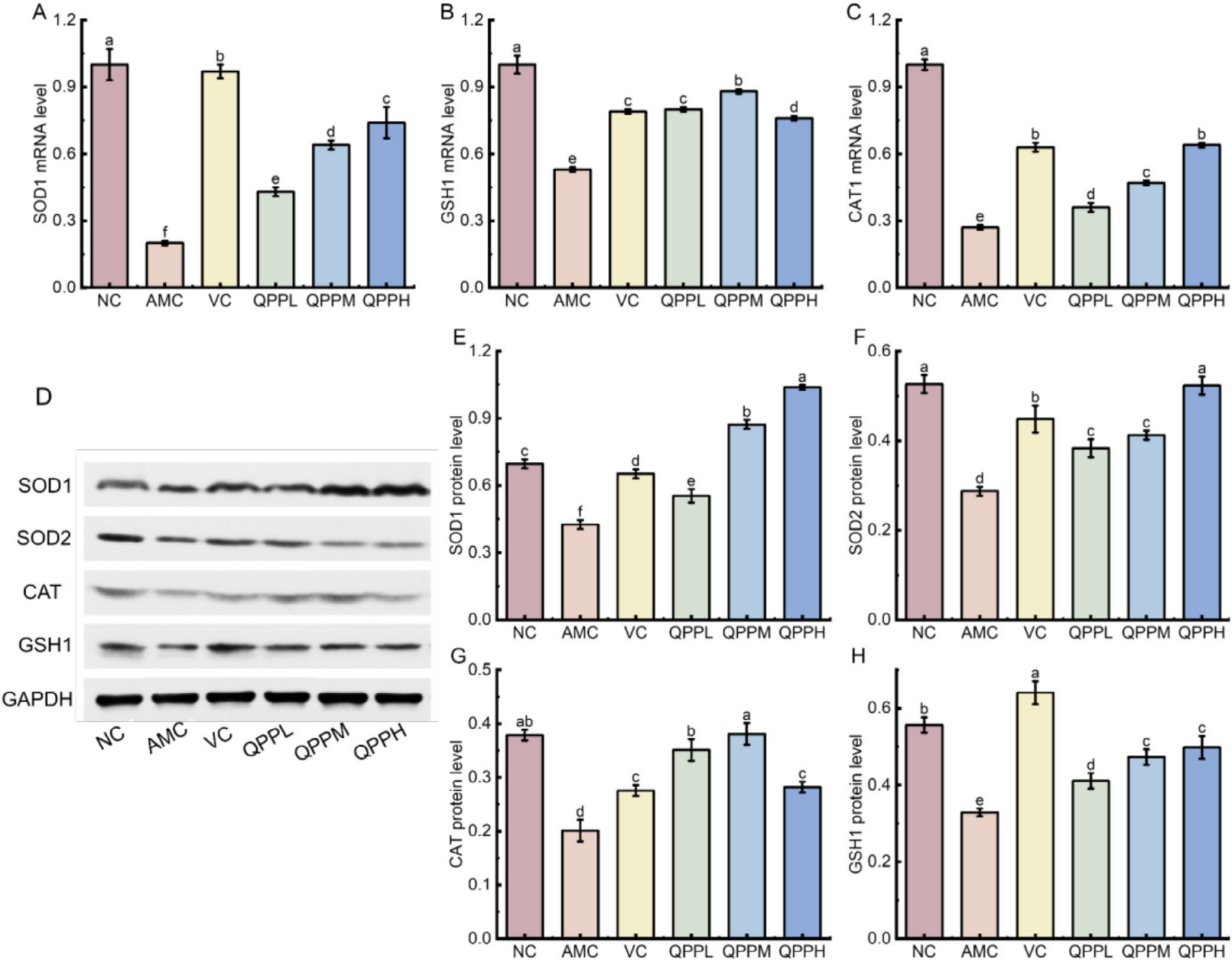
Effect of QPP on the relative expression levels of mRNA and protein for oxidative stress‐related genes in the liver. (A) SOD1 mRNA level; (B) GSH mRNA level; (C) CAT mRNA level; (D) Using GAPDH as a loading control, western blot analysis was used to determine the expression of (E) SOD1, (F) SOD2, (G) CAT, (H) GSH1. Data shown were the mean ± SD (*n* = 3).

As shown in Figure 7A, SOD1 expression was significantly lower in the AMC, VC, QPPL, QPPM, and QPPH groups compared to the NC group (*p* < 0.05), with the lowest relative expression observed in the AMC group. However, SOD1 levels in the VC, QPPL, QPPM, and QPPH groups were significantly higher than those in the AMC group. Furthermore, a clear dose‐dependent trend was evident across the treatment groups. These findings indicate that QPP can significantly upregulate SOD1 expression in the liver tissues of D‐gal‐induced aging mice.

As shown in Figure 7B, compared with the NC group, GSH1 expression was significantly reduced in the AMC group (*p* < 0.05), as well as in the VC group and QPP groups (*p* < 0.05). However, when compared to the AMC group, both the VC group and QPP groups exhibited higher levels of GSH1 expression (*p* < 0.05). These results indicate that QPP can significantly upregulate GSH1 expression in the liver tissue of D‐gal‐induced aging mice.

As shown in Figure 7C, compared with the NC group, the relative expression of CAT was significantly lower in the AMC, VC, and QPP groups (*p* < 0.05), with the lowest level observed in the AMC group. When compared to the AMC group, CAT expression was elevated in the VC, QPPL, QPPM, and QPPH groups (*p* < 0.05). Furthermore, a clear dose–response relationship was evident across the treatment groups. These findings indicate that QPP can significantly upregulate the relative expression of CAT in the liver tissue of D‐gal‐induced aging mice.

### 3.9 Effect of QPP on the Expression of SOD1, GSH1, and CAT Proteins in Mouse Liver

As shown in Figure 7D, representative Western blot bands of SOD1, SOD2, CAT, and GSH1 in mouse liver tissues are presented. In Figure 7E, compared with the NC group, SOD1 protein expression in the AMC group was significantly reduced (*p* < 0.05). However, SOD1 levels in the VC group and all QPP treatment groups were significantly higher than those in the AMC group (*p* < 0.05), with the QPPH group exhibiting the most pronounced increase, approaching or even surpassing NC group levels. As shown in Figure 7F, SOD2 protein expression in the AMC group was markedly lower than in the NC group (*p* < 0.05), whereas the VC and all QPP dose groups showed significantly elevated SOD2 levels relative to the AMC group (*p* < 0.05), with the greatest restoration observed in the QPPH group. In Figure 7G, CAT protein expression was significantly decreased in the AMC group compared to the NC group (*p* < 0.05); however, the VC, QPPL, QPPM, and QPPH groups displayed significantly upregulated CAT relative to the AMC group, with the QPPM and QPPH groups showing the most robust increases. As shown in Figure 7H, GSH1 protein expression was significantly reduced in the AMC group compared to the NC group (*p* < 0.05), despite a trend toward higher baseline levels in NC; the VC and all QPP‐treated groups exhibited significantly higher GSH1 expression than the AMC group (*p* < 0.05), with the QPPM and QPPH groups demonstrating the most substantial recovery, nearly reaching NC group levels.

In summary, D‐gal‐induced aging in mice was associated with a significant downregulation of key antioxidant proteins, including SOD1, SOD2, CAT, and GSH1. Treatment with VC or various doses of QPP, particularly the medium and high doses, effectively restored the expression levels of these proteins. Notably, the QPPH group exhibited the most pronounced upregulatory effects on the majority of the analyzed proteins, indicating that QPP intervention may exert its protective effects by enhancing the expression of endogenous antioxidant defense systems.

## 4 Discussion

Antioxidant peptides are naturally as inactive sequences within the primary structure of proteins (Lorenzo et al. 2018). These bioactive fragments can be released through enzymatic hydrolysis in vitro, microbial fermentation in food systems, or during postprandial protein degradation via autolysis and gastrointestinal digestion (Aderinola et al. 2019). The antioxidant capacity of protein hydrolysates is influenced by multiple factors, among which the specificity of the hydrolytic enzyme (Tacias‐Pascacio et al. 2020) and the DH (Rapalli et al. 2019) play pivotal roles. Both excessively high and low DH values may result in either inactive free amino acids or large low‐activity active peptides (Mardani et al. 2022). Neutralase, Papain, Alcalase, Pepsin, Flavourzyme, and Trypsin—each with distinct cleavage specificities along polypeptide chains—are capable of generating antioxidant peptides during the breakdown of natural proteins (Ren et al. 2021; Wang et al. 2021). However, the type and potency of the resulting peptides vary considerably depending on the enzyme's substrate preference and cleavage pattern. Notably, Pepsin preferentially cleaves peptide bonds adjacent to aromatic amino acids (e.g., Phe, Tyr, Trp) and acidic residues (e.g., Asp, Glu), both of which are well‐documented contributors to antioxidant activity due to their ability to scavenge free radicals through hydrogen donation or metal ion chelation (Matusiewicz et al. 2025; Serrano‐Sandoval et al. 2023). Moreover, Pepsin achieves an optimal balance between peptide yield and DH after 3 h of hydrolysis: this condition minimizes the generation of inactive free amino acids (associated with excessive DH) while promoting the release of soluble, bioactive peptides—properties essential for effective antioxidant function. In this study, quinoa protein was hydrolyzed using the six aforementioned proteases. Consistent with these mechanistic advantages, the 3 h Pepsin‐derived hydrolysate demonstrated the highest free radical scavenging activity compared to hydrolysates generated by other enzymes.

In addition, the antioxidant activity of protein hydrolysates should be comprehensively evaluated using multiple in vitro assays that operate through distinct mechanisms and in different reaction environments. To assess the antioxidant properties of QPP, several well‐established methods were employed. The DPPH radical scavenging assay is widely used to evaluate the free radical neutralizing capacity of antioxidant due to its high sensitivity and rapid reaction kinetics (Li et al. 2018). Superoxide radicals, another harmful ROS, can initiate the formation of hydroxyl radicals—the most reactive and damaging ROS—leading to oxidative stress and cellular dysfunction. The PFRAP assay measures antioxidant activity based on the reduction of Fe (III) to Fe (II), which subsequently forms a Prussian blue complex; the intensity of the resulting deep blue color is quantified spectrophotometrically. Unlike radical‐based assays, PFRAP does not involve free radicals, thereby serving as a complementary method for assessing reducing capacity as an indicator of antioxidant potential (Munteanu and Apetrei 2021). As shown in Figures 1 and 2, although the DH induced by Pepsin was not the highest among the tested enzymes, the corresponding hydrolysate demonstrated the strongest in vitro antioxidant activity across multiple assays. Therefore, Pepsin was selected as the proteolytic enzyme for subsequent experiments.

Amino acid composition and hydrophobicity are well‐recognized critical factors influencing the free radical‐scavenging activities of antioxidant peptides (Bo et al. 2021). As demonstrated in Table 3, pepsin hydrolysis dramatically reshaped the amino acid profile of quinoa protein, leading to substantial enrichment of several functionally active residues. Notably, the potent antioxidant activity of QPP can be mechanistically attributed to the significant enrichment of four key residues: Tyr, His, Phe, and Lys (Kim et al. 2020). The phenolic hydroxyl group of Tyr, whose content increased from 30.63 to 865.46 mg/mL, approximately 28‐fold, enables effective hydrogen atom donation to neutralize free radicals, which is fundamental to the observed DPPH scavenging capacities (Blanca et al. 2005). Concurrently, His levels rose from 15.86 to 416.17 mg/mL, roughly 26‐fold. The imidazole ring of His contributes to both metal ion chelation and free radical stabilization, thereby mitigating oxidative stress (Jiang et al. 2019). The most pronounced increase was observed for Phe, which rose from 29.85 to 1315.24 mg/mL, nearly 44‐fold. The hydrophobic and aromatic nature of Phe enhances peptide‐lipid interactions, facilitating access to lipid‐soluble radicals and supporting radical quenching. In addition, Lys content increased from 29.99 to 827.46 mg/mL, about 28‐fold. The positive charge of Lys significantly improves the water solubility of peptides, enhancing their bioavailability and access to reactive species (Matsui et al. 2018). Collectively, the dramatic enrichment of these four functionally active residues, rather than a simple increase in total amino acid content, provides a clear mechanistic basis for the superior antioxidant activity of QPP. Their unique structural properties, including hydrogen donation, metal chelation, hydrophobic interaction, and solubility enhancement, act synergistically to confer potent free radical‐scavenging effects.

The D‐gal‐induced aging model has been widely used to mimic oxidative stress‐related pathological changes characterized by excessive reactive oxygen species accumulation, impaired antioxidant systems, and accelerated tissue damage. In the present study, chronic D‐gal administration resulted in lower body weight gain, decreased activities of SOD, GSH‐Px, and T‐AOC, as well as increased MDA content (Azman and Zakaria 2019; Lan et al. 2023; Li et al. 2019), confirming the successful establishment of the aging model.

The anti‐aging effect of QPP is closely associated with its potent antioxidant capacity. In the present study, QPP effectively mitigated D‐gal‐induced physiological disorders including the decline in body weight gain and significantly restored the antioxidant system by enhancing SOD, GSH‐Px, and T‐AOC activities while reducing MDA accumulation. As typical and widely accepted indicators for evaluating oxidative stress and aging progression in in vivo models, these results clearly demonstrate that QPP can alleviate oxidative damage and delay aging‐related physiological deterioration.

Although key aging biomarkers and related signaling pathways were not evaluated in the current study, the whole‐animal model used here has higher physiological relevance than in vitro cell systems. The observed improvements in systemic antioxidant status and overall aging phenotypes provided reliable evidence supporting the anti‐aging potential of QPP. The enhanced antioxidant activity mediated by QPP may protect tissues from oxidative injury, thereby contributing to its beneficial effects against D‐galactose‐induced aging.

As shown in Figure 3A, mice subjected to subcutaneous D‐gal administration for 10 weeks exhibited aging‐related phenotypic abnormalities, including reduced weight gain, decreased locomotor activity, delayed responses, and hair loss. Impaired immune function is a well‐established contributor to the aging process, and thus immune parameters serve as key biomarkers for evaluating the extent of aging. In this study, the D‐gal‐induced group showed significantly reduced thymus and spleen indices (Figure 3C,D), indicative of immune system dysfunction. However, QPP treatment effectively ameliorated these phenotypic signs of aging and restored thymus and spleen indices toward normal levels. Given that immune dysfunction accelerates aging progression, these findings suggest that QPP may exert protective effects against organ aging by enhancing immune function.

Under normal physiological conditions, the body's oxidative and antioxidant systems maintain a state of dynamic equilibrium. Endogenous antioxidant enzymes play a critical role in neutralizing free radicals and maintaining redox homeostasis. However, when exposed to external stressors that induce oxidative stress, metabolic balance is disrupted, and the body fails to sustain its initial equilibrium. Lipid peroxidation ensues, leading to the accumulation of reactive peroxidation products. Extensive evidence indicates that the balance between the ROS system and the antioxidant defense system is a key determinant of the extent of oxidative stress (Martel et al. 2020). In this study, we found that the activities of antioxidant enzymes (SOD, GSH‐Px, and CAT) and T‐AOC in both liver tissue and serum were significantly reduced in the AMC group compared to the NC group. Conversely, the levels of MDA (a major peroxidation product) were markedly elevated in the AMC group, as shown in Figure 4, indicating a significant disruption of the antioxidant system. T‐AOC reflects the cumulative capacity of both enzymatic and non‐enzymatic antioxidants to scavenge ROS, making it a reliable indicator of overall redox status (Wu et al. 2019). MDA, as a terminal metabolite of lipid peroxidation, serves as a sensitive marker for assessing the degree of oxidative damage in tissues. Once released from damaged cell membranes, MDA can react with proteins and nucleic acids, inducing cross‐linking and polymerization, and may also inhibit protein synthesis. These effects are primarily mediated through structural and functional impairment of cellular membranes, including altered permeability, which subsequently interferes with normal biochemical antioxidant processes.

Additionally, excessive production of ROS in biological systems can induce oxidative damage to tissues, impair membrane integrity, disrupt cellular metabolism, and lead to the inactivation of proteins and enzymes, particularly in organs with high metabolic rates such as the liver, kidneys, and lungs. The elevated accumulation of MDA in the livers of AMC group mice further corroborates this observation. Histological examination revealed increased inflammatory cell infiltration, hepatocyte necrosis, glomerular atrophy, and alveolar cell necrosis in the AMC group, whereas treatment with QPP or VC partially ameliorated the structural damage to the liver, kidneys, and lungs caused by chronic D‐gal administration (Figure 5). These histopathological findings were highly consistent with the results of biochemical analysis. Furthermore, VC exhibited antioxidant effects, primarily by enhancing the activity of antioxidant enzymes in mouse livers, reducing MDA levels, and providing a degree of protection against hepatic dysfunction. TEM analysis of liver tissue demonstrated that QPP exerts protective and reparative effects on hepatocytes by preserving overall cellular architecture and restoring the ultrastructural morphology of key organelles, including mitochondria and endoplasmic reticulum. Notably, the QPPH group displayed morphological features closer to those observed under normal physiological conditions (Figure 6). Therefore, it can be concluded that QPP may mitigate D‐gal‐induced structural and functional impairments in the liver by restoring antioxidant enzyme activity and reducing lipid peroxidation levels, while also exerting a suppressive effect on oxidative stress in hepatocytes.

SOD is a crucial enzyme in the human antioxidant defense system. Depending on its bound metal cofactors, SOD is classified into two primary isoforms: SOD1 (Cu/Zn‐SOD), located in the cytosol of blood cells and various tissues, and SOD2 (Mn‐SOD), which resides in the mitochondrial matrix (Fukai and Ushio‐Fukai 2011). SOD catalyzes the dismutation of excess superoxide anion radicals into H_2_O_2_, which is subsequently detoxified by CAT and GSH‐Px into H_2_O, thereby protecting cells from oxidative damage. With aging, SOD activity progressively declines (Li et al. 2019), leading to an accumulation of reactive oxygen species and accelerated lipid peroxidation. To investigate the molecular mechanisms underlying QPP's effects, we examined both the transcriptional levels of SOD1, GSH1, and CAT and the protein expression levels of SOD1, SOD2, CAT, and GSH1. Our results showed that in D‐gal‐induced aging mice, QPP treatment upregulated the mRNA expression of SOD1, CAT, and GSH1 in liver tissue, accompanied by increased protein levels of SOD1, SOD2, CAT, and GSH1 (Figure 7). These findings indicate that the anti‐aging effect of QPP is associated with the enhanced expression of key antioxidant enzymes, including SOD1, SOD2, CAT, and GSH1.

## 5 Conclusions

This study evaluated protease selection for quinoa protein hydrolysis and the anti‐aging activity of its peptides. Among six proteases (Neutralase, Papain, Alcalase, Pepsin, Flavourzyme, Trypsin), pepsin was optimal: it achieved balanced DH to avoid inactive products, and its derived peptides (QPP) were enriched in Leu, Phe, Arg—amino acids enhancing antioxidant activity. In D‐gal‐induced aging mice, QPP reversed thymus and spleen atrophy, alleviated pathological damage in the liver and kidneys, increased the activities of SOD, CAT, GSH‐Px, and T‐AOC, and decreased MDA levels in serum and liver (all *p* < 0.05). Molecularly, QPP upregulated the expression of antioxidant genes (SOD1, GSH1, CAT) and proteins (SOD1, SOD2, CAT, GSH1) in the liver, confirming that QPP enhances the endogenous antioxidant defense system. This study supports pepsin for bioactive QPP preparation and identifies QPP as a promising natural anti‐aging ingredient for functional foods.

## Author Contributions

**Dandan Gao:** funding acquisition, writing – review and editing, validation, supervision, resources. **Hongxin Ma:** validation, software, methodology. **Yuxuan Zhang:** writing – original draft, visualization, software, methodology, investigation.

## Funding

This study was financially supported by the Key Research and Development Project of Gansu Province (24YFFA026); the Industry Support Program Project of Universities and Colleges in Gansu Province (2026CYZC‐009).

## Conflicts of Interest

The authors declare no conflicts of interest.

## Data Availability

The data that support the findings of this study are available from the corresponding author upon reasonable request.
